# Virtual Reality Meets Diabetes

**DOI:** 10.1177/19322968231222022

**Published:** 2024-01-09

**Authors:** Neil Vaughan

**Affiliations:** 1 Department of Clinical and Biomedical Science, NIHR Exeter Biomedical Research Centre, Exeter Centre of Excellence in Diabetes, University of Exeter, Exeter, UK; 2 Royal Academy of Engineering, London, UK

**Keywords:** virtual reality, augmented reality, diabetes, training

## Abstract

**Background:**

This article provides a detailed summary of virtual reality (VR) and augmented reality (AR) applications in diabetes. The purpose of this comparative review is to identify application areas, direction and provide foundation for future virtual reality tools in diabetes.

**Method:**

Features and benefits of each VR diabetes application are compared and discussed, following a thorough review of literature on virtual reality for diabetes using multiple databases. The weaknesses of existing VR applications are discussed and their strengths identified so that these can be carried forward. A novel virtual reality diabetes tool prototype is also developed and presented.

**Results:**

This research identifies three major categories where VR is being used in diabetes: education, prevention and treatment. Within diabetes education, there are three target groups: clinicians, adults with diabetes and children with diabetes. Both VR and AR have shown benefits in areas of Type 1 and Type 2 diabetes.

**Conclusions:**

Virtual reality and augmented reality in diabetes have demonstrated potential to enhance training of diabetologists and enhance education, prevention and treatment for adults and children with Type 1 or Type 2 diabetes. Future research can continually build on virtual and augmented reality diabetes applications by integrating wide stakeholder inputs and diverse digital platforms. Several areas of VR diabetes are in early stages, with advantages and opportunities. Further VR diabetes innovations are encouraging to enhance training, management and treatment of diabetes.

## Introduction

Diabetes is becoming increasingly common and affects approximately 463 million people worldwide (8.8% of the adult population), according to the International Diabetes Federation (IDF) Atlas.^1^ Type 1 diabetes (T1D) affects around 10% of those and Type 2 diabetes (T2D) affects the other 90%. Diabetes doubles a person’s risk of early death according to the World Health Organization (WHO)^2^ and resulted in 4.2 million deaths in 2019.^1^

Virtual reality (VR) is recently rapidly increasing in popularity with the decreasing cost of headsets, VR is now affordable for home and health care use. Advantages of VR health care are increasingly established, as highlighted by the Medicine Meets Virtual Reality (MMVR) series, including orthopedics,^3,4^ anesthetics,^5,6^ paramedics,^7,8^ resuscitation,^9^ medical examination scoring^4,5,10^ amongst many others. Digital twin technology is setting a wave of transformations in health care.^11^ Digital twin concept is closely linked with VR as a major platform in which health care aspects can be integrated,^12^ with potential for diabetes.^13^ Smart health care applications increasingly use Digital Twin–powered artificial intelligence (AI) with medical devices^14^ as part of the current virtual revolution. Digital twins are a 2023 strategic priority for the Alan Turing Institute (ATI) and an area of research and innovation strength. In 2023, at AI UK, was the launch of Turing Research and Innovation Cluster in Digital Twins (TRIC-DT).

For diabetes training, VR is particularly suitable, because the physiological characteristics are especially responsive to patients’ lifestyle, physical and cognitive change.^15^ Diabetes care is being continually digitized as the pace of people getting diabetes is growing faster than the growth in number of diabetologists,^15^ further highlighting VR training as key to help people with diabetes improve self-management.

Overall, this research has found that VR and augmented reality (AR) in diabetes have been associated with three main areas in which applications of VR have been developed for diabetes: education, prevention, and treatment. (1) Education and training in diabetes using VR applies to three target groups: clinicians, adults with diabetes, and children with diabetes. (2) Prevention of diabetes complications with VR is achieved by encouraging exercise, healthy behavior, stress reduction, and healthy eating choices. (3) Treatment and management of diabetes with VR includes augmented carbohydrate estimation, tracking and guiding insulin injections, and virtual clinical consultations. The following sections of this review have been divided into those three major categories.

### Conventional Training in Diabetes Without VR

For people with T1D aged over 17, in the UK, the NHS provide conventional diabetes training without VR, through Dose Adjustment For Normal Eating (DAFNE).^16^ The DAFNE training module aims to train people with diabetes to (1) achieve more glucose checks in target without increasing the risk of severe hypoglycaemia; (2) reduce negative impacts of diabetes including anxiety, stress, and time spent at NHS appointments; and (3) get the best results from insulin therapy. The DAFNE training modules have potential to benefit from being supplemented by VR for improved simulation and immersion.

### Search Criteria

All major databases of literature were searched, including PubMed, IEEE Xplore, Google scholar, ASME Digital library, Web of Science, EMBASE, EBSCO Host and Cochrane database of clinical trials. Keywords “Virtual Reality,” “Diabetes,” and “Training” were used.

The years of search were set to 2019 to 2023. Previous articles have also been included by tracing references in related areas in the returned matching result papers.

## VR in Diabetes

This section details uses of VR in diabetes, within three major categories: Education (for clinicians, adults, and children), Prevention, and Treatment.

### VR Diabetes Education for Clinicians

Within education and training for diabetes, there are various circumstances in which difficulties emerge where VR can be of help. Virtual reality can help with training health workers in rural areas, where there is often a lack of diabetes experts and the prevalence of diabetes can be almost twice the national average, such as in Appalachian Ohio.^17^

**Figure 1.**
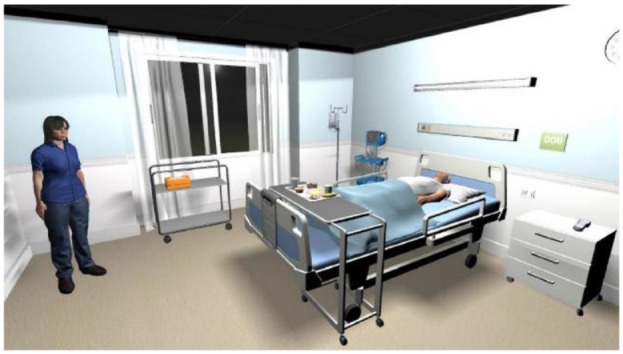
Virtual clinical training was found superior to normative training when used for education of second-year nursing students (n = 171),^18^ reproduced with permission of CC-BY license.

A trial^18^ evaluated the use of VR for diabetes training, with 171 second-year nursing students. This found that VR was significantly (*P* ≤ .001) better for teaching T2D hypoglycemia knowledge than normative methods, due to increased engagement and immersion.^18^ The VR training simulator for this study was created by Daden Ltd., using Unity 3D software (Figure 1). The clinical scenario involves a replica of a local hospital room, with nurse and patient avatars. The simulation begins with a handover providing patient information. Later, the patient deteriorates and experiences hypoglycaemia and the aim is for students to correctly identify and treat this condition.

A VR diabetes training system for clinicians was developed by Oxford Medical Simulation (OMS) with University Hospital Southampton (UHS) and Portsmouth Hospital^19^ in partnership with NHS England Diabetes Team as part of The NHS VR Diabetes Initiative. The training was initially planned during the COVID-19 pandemic to be delivered for free by the OMS team to assist with socially distanced training. Oxford Medical Simulation collaborated with University Hospital Southampton^20^, to develop immersive VR diabetes scenarios for clinicians. This provides clinicians with safe practice diabetes emergencies, aiming to improve care for diabetes patients during hospital admissions. The system uses Oculus Rift VR headset and gives personalized feedback. The VR aims to improve clinical decision-making under pressure, crisis resource management, team interaction, and patient engagement. This was funded by an educational grant from Novo Nordisk^20^ and trialed across the south of England in partnership with Health Education England (HEE), piloted with 10 doctors. After completing VR diabetes education, a curriculum mapped certificate for their ePortfolio was sent to the clinicians who completed the training. The OMS diabetes VR project, titled “Diabetes Emergencies: Virtual Interactive Clinical Education (DEVICE)” was evaluated in a trial by^21^ with 39 second-year junior clinicians from two U.K. hospitals (University Hospital Southampton and Queen Alexandra Hospital, Portsmouth) using Kirkpatrick’s training model. The trial found that after using VR, confidence increased in diabetes emergency management. Amongst trainees, 100% found the scenarios were suitable for their level, 72% expected they would use the knowledge within a week and 28% had increased confidence in managing Diabetic Ketoacidosis (DKA) after using VR. This suggests that VR is a useful, popular educational tool. One weakness was that delivering the VR experience is a fairly time-consuming exercise as only one trainee could use the system at a time.

A VR training program for clinicians has been designed for use in rural areas of Ohio which have nearly double the national average diabetes prevalence rate. This aims to improve cultural self-efficacy and diabetes attitudes, by training the primary care workforce on new emerging therapies for T2D,^17^ to meet rising remands due to the lack of certified diabetes experts in rural areas. Evaluation amongst clinicians (n = 69) found improvements in all three of the cultural self-efficacy subscales: Cognitive, Practical, and Affective, finding that VR for diabetes training is an innovative approach to improve cultural self-efficacy and diabetes attitudes.

Virtual reality is used for diabetes as part of Simulation-Based Medical Education (SBME) which supports the development of competencies in diabetes technical skills and human factors (or nontechnical skills) and is an excellent tool to allow staff prepare for clinical diabetes crises that are seen every day in health care.^21^

Virtual reality medical training has proven useful during pandemics such as COVID-19 to enable remote health care training for clinicians while maintaining social distancing.

A VR system has been proposed for training in the diagnosis and treatment of Diabetic Foot.^22^ The system uses a VR simulator for training students and professionals in primary health care. The proposed VR system will use Blender 3D, Unity, and Oculus Quest headset.

### VR Education for Adults With Diabetes

In rural areas, VR training for adults and children with diabetes can be particularly useful as rural areas tend to have lower health literacy, higher chance of delayed diagnosis,^17^ and limited availability of diabetologists, despite an above average prevalence rate of diabetes.

**Figure 2.**
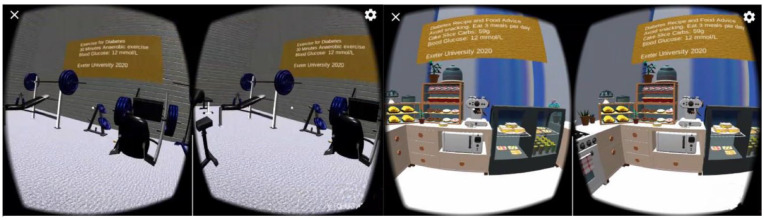
The virtual reality diabetes training platform for people with diabetes developed by the author at the University of Exeter. (Left) a VR gymnasium for exercise with diabetes, (Right) a VR kitchen with diabetes nutrition advice.

At the University of Exeter, the author has developed a prototype VR diabetes training platform for people with diabetes. This contains various scenarios including training for people with T1D to exercise safely avoiding hypoglycaemia in a VR gymnasium, food carbohydrate counting advice in a VR kitchen (Figure 2), newly diagnosed training for finger prick and a scenario for glucose monitoring at the workplace. Input and feedback was received from clinicians at Royal Devon University Healthcare NHS Foundation Trust in Exeter, United Kingdom and Taunton Musgrove Park Hospital. This VR diabetes training platform has been tested on Android smartphones using Google cardboard and Windows PC platform with VR head-mounted display (HMD).

**Figure 3.**
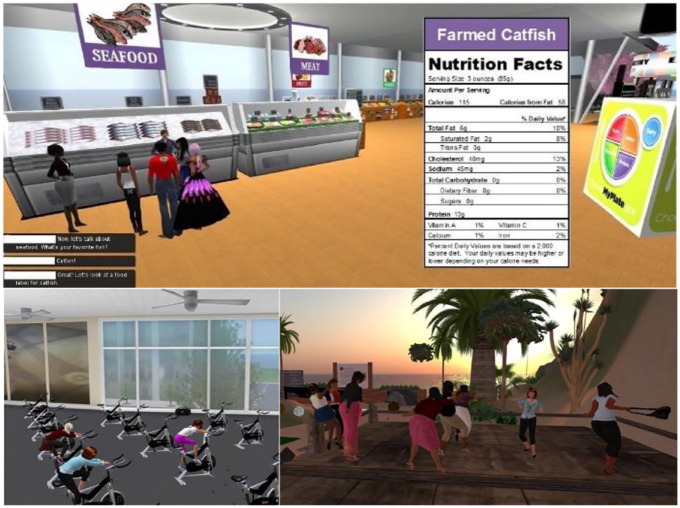
(Upper) Education sessions on physical activity and healthy eating with diabetes.^23^ (Lower left) Virtual exercise facilities for indoor cycling and (Lower right) beach dancing stage for virtual reality diabetes exercise training,^24^ reproduced with permission of CC-BY license.

The American Diabetes Association (ADA)’s written training materials and videos for diabetes have been integrated into a virtual learning center^23^ to deliver training within a VR environment built in “Second Life” (SL) virtual world (VW; Figure 3, upper). This also integrates 3D images with tips on label reading, virtual avatars of exercise researchers, health professionals, and dietitians, to present 10 education sessions on physical activity and healthy eating with diabetes. The VR intervention resulted in a significant increase in real-life physical activity levels and a decrease in dietary meat intake.

A VW in SL, was developed by Rosal et al,^24^ for diabetes education (Figure 3, lower right). The education is targeted toward African American women, because this group report lowest levels of physical activity, and 66% of African Americans with diabetes have high-fat diets. Evaluation found that self-management education for T2D can be delivered using virtual exercise facilities and food exhibits. The VR system was used to deliver the evidence-based behavior-change curriculum, which seeks to enhance diabetes knowledge. It also aims to optimize attitudes toward diabetes self-management, develop behavioral self-management skills including glucose tracking and goal setting, facilitate changes in dietary intake, physical activity, blood glucose self-monitoring, and medication adherence.^24^ The VR diabetes education was compared to in-person format,^24^ finding that physical activity increased by 18% in the virtual group, whereas it decreased by 25% in the face-to-face group.

Sense of presence in VR can lead to greater engagement and attention, and the use of VWs has been shown to influence behavior in the real world,^24^ which can be of benefit to people with diabetes implementing lifestyle changes.

Educational games for diabetes have been developed on Nintendo Wii Fit Plus for adults with diabetes and the evaluation found Wii Fit successfully increased physical activity levels in the intervention group.^25^

By 2030, the use of VR for diabetes education is expected to include insulin initiation and diabetes device training.^15^

### VR Education for Children With Diabetes

Nutrition education for children with diabetes is more effective when delivered using interactive multimedia methods such as VR.^26^ Virtual reality leads to improved levels of HbA1c in children and improves communication aspects.^26^ However, VR diabetes training should be conducted at frequent intervals due to transient effect of education.

Children need an engaging method to learn about diabetes conditions, and VR can provide a way to integrate health care advice into engaging virtual diabetes game content for children. Virtual reality is useful for integrating gamification to support diabetes education,^15^ and more than 542,000 children in the world are living with T1D.

A virtual serious game for young children with T1D^27^ was developed using an Intelligent Agent with virtual coaching strategy using a doctor avatar’s knowledge base about diabetes interventions to suggest best actions are correct behaviors. Evaluation showed that this allows basic concepts and skill about diabetes to be acquired, demonstrating learning effectiveness and the appreciation of young patients and parents. Digital games for teaching about T1D and T2D can help children, adolescents, and adults with diabetes to better cope with their lifelong condition.^28^ This demonstrates the potential of diabetes VR integrating gamification and social in-game components, to motivate and educate patients to positively change behavior and lifestyle.

**Figure 4.**
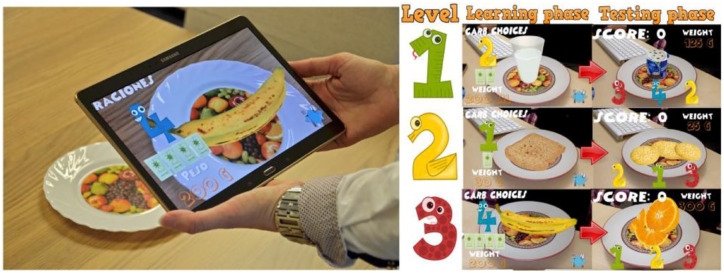
Augmented reality carbohydrate training for children with diabetes,^29^ reproduced with permission of CC-BY license.

For carbohydrate content estimation, AR has been used to train children^29^ using a therapeutic education AR game where the carbohydrate contents of the food are overlaid onto the video feed of a real plate of food (Figure 4). Evaluation confirmed that (1) children acquired new knowledge about carb choices from the AR game, (2) another benefit of AR training for diabetes is that it is versatile and can be performed at any place and time, and (3) the pervasive educational AR game has great potential for therapeutic education in diabetes.

Virtual diabetes care simulations can help to learn and improve self-management and treatment,^30^ by performing virtual diabetes care, partaking in quizzes of diabetes-related knowledge, using their own treatment data and also include social media competing with other players.

Virtual worlds and virtual avatars have often been used within games for diabetes education. A VW game was developed to benefit children with T1D,^31^ suggesting that virtual games should include these three key aspects: (1) identify oneself with the game’s main character, (2) report/support the player’s self-care, and (3) provide information about the underlying biochemical mechanisms that trigger the symptoms of the disease and provide the rationale for healthy behavior.

To improve self-care behavior in children with T1D, the video game Packy & Marlon was developed by WaveQuest as an educational platform and published by Raya Systems for the Super Nintendo Entertainment System (SNES). Studies found that playing the game helped improve communication about diabetes between children and parents, improve children’s self-care behaviors and reduce the number of emergencies.^32^

**Figure 5.**
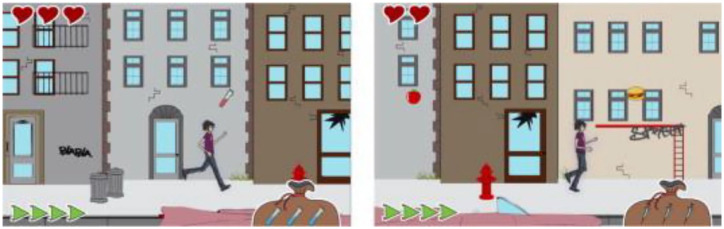
Balance, a 2D virtual diabetes platform game where the player can run and jump whilst monitoring blood glucose, taking insulin injections, and choosing foods, created by Fuchslocher et al,^33^ reproduced with permission from RightsLink Elsevier.

Virtual games for children’s diabetes education include Balance^33^ (Figure 5) and the Android game Huima Hiilari (HUS-kuntayhtm, 2017), in which the player can jump and run whilst monitoring blood glucose, taking insulin injections, and choosing foods. Other diabetes games include Jerry the Bear (Sproutel Inc., 2019) and Dex: Your Virtual Pet (Augusta University, 2016) which enable children to play whilst learning more about diabetes and how to make healthy food choices, by feeding and treating the virtual pet with food or drugs to improve their condition. In the game Diapets (Giancarlo Cavalcante, 2016) children learn about T1D by treating the virtual game character and the game synchronizes with Apple HealthKit framework to obtain the player’s activity data and support self care. The game Commander Gage: T-1 Space Rangers (Fletcher/Rosen Interactive, 2016) requires the player to search and mine for life-saving insulin.

The Diabetic’s Diner game (iWOO Health, 2016) entails virtual dietary challenges and selection of healthy food items. Android games BuildupBlocks and CarboBuster (Mahalo Health Inc., 2019),^34^ involve quizzes and puzzles to learn about carbohydrate content of foods. HealthSeeker (Diabetes hands foundation [DHF]) is a game that encourages diabetes management and health improvement, connected with social media allowing Facebook friends to see achievements. Diabattle (N8 Solucoes Inteligentes, Ltda., 2019), is a game encouraging children to take care of their own health through daily diabetes activities (blood glucose measurements, insulin intakes, counting carbohydrates and exercise) and can also visualize blood glucose results for the last day, week, or month.

The Nintendo Wii Fit Plus has been found effective among children with T1D to encourage movement and exercise.^25^ Virtual reality and AR also have been useful to explain to children the purpose of taking insulin, which makes it more likely that children will be capable of self-managing their condition.

#### VR for preventing diabetes complications

Virtual reality was used in prevention^35^ of further complications in people with T2D,^36,37^ by encouraging and motivating exercise and to combat depression. These VR games aimed to motivate exercise and combat depression to reduce the influence of T2D on patients’ minds and bodies. These systems showed that VR gamification was useful in the health care system, helping to prevent further detrimental effects of T2D, by motivating users to exercise and healthy life choices. This was evaluated with 30 participants^37^ and helped prevent some consequences of T2D.

In other areas outside of diabetes, VR has been used for the prevention of other diseases, such as by encouraging exercise, healthy behavior training, and to improve stress, mental and psychological conditions such as Alzheimer’s and stroke.^35^ Some of these VR aspects could potentially be translated to prevention of diabetes.

#### VR for treating or managing diabetes

For managing diabetes, VR can help in various scenarios. Virtual reality has a strong potential for positive impact on diabetes self-management,^15^ and it can help facilitate weight loss.^38^ Virtual reality could be particularly useful for managing diabetes for insulin-treated individuals in how to deal with common challenging situations such as physical activity and exercise,^15^ long airline flights or preparing for elective hospital procedures.^15^

Virtual reality diabetes education has shown advantages in improving eating habits^15^ and to facilitate weight loss.^38^

By 2030, the use of VR for diabetes management is expected to expand to include improving eating habits, facilitating weight loss, increasing physical activity and other lifestyle changes.^15^

When people are considering meals or diet, AR can help to identify the food’s effect on blood sugar and the glycemic index of food.^39^ An AR food volume estimation was developed,^40^ using the user’s smartphone to create a 3D volume estimation overlaid onto food, the AR system can provide nutrition information which is automatically computed using AI.

Augmented reality tools have been used to assess improvements in the accuracy of carbohydrate counting for people with T1D.^41^ This AR system has been combined with using a smartphone to log physical activities, blood glucose, and insulin. The AR system significantly improved carbohydrate counting accuracy. Some disadvantages for AR carb counting include complexity and time taken to draw foods, and AR carb counting was less effective for meals containing multiple mixed foods.

Augmented reality can also help diabetes in areas of encouraging exercise programs, foot maintenance, and capillary glucose measurement.^39^

Augmented reality could be used to pinpoint where on the body insulin should be injected, and to also keep a log of where insulin was injected in the recent past to help avoid lipoatrophy.^39^ These are helpful features for both newly diagnosed diabetics and those caring for diabetic loved ones. Augmented reality could show patients how much insulin to inject to avoid an accidental overdose that is a common cause of potentially fatal hypoglycaemia.^39^

Webcam consultations for diabetes^42^ have reported success, and VR could be a possible tool to further enhance remote interaction between clinician and patient, including virtual health sessions with individuals or groups.^43^

In other areas of health, VR treatments have been developed including: rehabilitation for walking gait or after stroke, physiotherapy for multiple sclerosois, adjuvant therapy such as pain distraction.^35^ Some of the features from these could be translated to benefit diabetes.

The use of augmented and VR to support diabetes self-management has demonstrated potential as another opportunity, through automation and immersion, however, they are still in their infancy and individuals should be adequately trained in the use of these technologies.^25^

## Comparative Assessment

The VR simulators for diabetes generally fit into three categories: education, prevention, and treatment. There has been evidence of benefits from VR in each of these categories. Within the education category, there are subcategories for clinicians, adults, and children. Table 1 highlights 15 recent VR diabetes applications in those categories. The majority of applications, twelve use VR as opposed to three that use AR. There is a lot of variation between the benefits of each VR model, for example, within clinical education, some focus on training diabetic foot^22^ whereas others focus on hypoglycaemia knowledge.^18^ The benefits of using VR/AR have been positively evaluated within the majority of categories.

**Table 1.** Comparative Assessment Between All Categories of VR Applications for Diabetes.

Category	Target	Reference	Evaluation	Benefits	VR/AR	Hardware
Education	Clinicians	Singleton et al^18^	171 students	Improved hypoglycaemia knowledge	VR	Unity 3D graphics VR platform with avatars on screen (Figure 1)
		Riva et al^22^	None	Diabetic foot training for student and clinicians	VR	Unity 3D VR avatars with Blender 3D, hand tracking on Oculus Quest 2 headset, ray-casting hand touch control.
		Beverly et al^17^	69 participants, mean age 42 years	Improved rural self-efficacy, diabetes attitudes	VR	360° videos (cine-VR) VR simulation
		Beverly et al^20^	39 clinicians, increased DKA confidence, positive feedback on immersive teaching	Improved self-confidence in diabetes emergencies	VR	Oculus Rift VR 360
	Adults with diabetes	Ahmadvand^44^	160 consumer panel, 17 participants + six survey response	Improve health literacy for T2D	VR	Smartphone-based AR system. Augment video onto factsheets.
		Ruggiero et al^23^	41 African Americans	Integrates ADA guidelines into VR training	VR	Second Life 3D Virtual World with avatars, interactive touch-screen with audio headset. (Figure 3, upper)
		Rosal et al^24^	100 African-American women, 18% more exercise	Self-management exercise and food	VR	Second Life 3D virtual world (Figure 3, lower)
	Children with diabetes	Pesare et al^27^	20 children aged 8-12. significant knowledge improvement between pretest and posttest.	Intelligent agent for diabetes concepts	VR	Client-server web application using knowledge base, inference engine and user interface. Virtual coach game simulation.
		Calle-Bustos et al^29^	70 children aged 5-14 with diabetes. Improved knowledge of carb choices.	AR Carb Estimation—virtual food on a real dish	AR	Mobile device, Android Operating System, device can zoom, rotate, raise, lower, or move in 3D. Requires images printed on paper.
		Lauritzen et al^30^	None	VR diabetes knowledge social media	VR	Diabetes Counseling, Avatar and mixed reality.
		Burkholz et al^31^	None	Diabetes symptoms and healthy choice rationale	VR	Smart device game, Unity, 3D virtual world, speech recognition
Prevention	People with diabetes	Neira-Tovar et al^36,37^	30 participants	Motivate exercise, combat depression	VR	Virtual World, Augmented reality glasses, oculus rift, Kinect 2.0, Unreal engine, Unity3D.
Treatment	People with diabetes	ARinMed^39^	None	Insulin location, log and dose	VR	Diabetes App smartphone based.
		Domhardt et al^41^	Eight adults, improved carb estimation	AR carb estimation	AR	AR food 3D model on smartphone screen
		Stütz et al^40^	28 users, significantly improves nutrition assessment	Meal volume and nutrition estimation	AR	AR food 3D model on smartphone screen. Unity 3D and Qualcomm Vuforia

Abbreviations: VR, virtual reality; AR, augmented reality; DKA, diabetic ketoacidosis; T2D, type diabetes; ADA, American Diabetes Association.

## Conclusion

Overall, this article provides an overview of how VR is currently being used for diabetes. This includes VR being used for training (of clinicians, adults and children), prevention, and treatment. The evaluations suggest that VR has demonstrated useful benefits in the majority of these categories.

Both VR and AR have shown benefits in areas of diabetes. The recent increase in availability of VR headsets such as Oculus Quest, Oculus Go, Google Cardboard, Sony PlayStation VR, and Samsung Gear VR has increased opportunities for clinicians and patients to be involved with development and evaluation of VR systems for training and management of diabetes.

The VR diabetes training for clinicians are not intended to replace the conventional training that clinicians receive but can offer an additional range of features and scenarios without risk to patients to supplement training. Diabetes VR has been used to train clinicians on diagnosis, prevention, and treatment.^22^ Diabetes VR has been useful training for clinicians in Diabetic Foot.^22^ For training clinicians on diabetes, VR training has outperformed normative training in terms of second-year nursing students knowledge of T2D hypoglycaemia knowledge.^18^ Diabetes VR training has been useful in rural areas, to meet higher demand due to above-average diabetes prevalence but fewer certified diabetes experts.^17^ Hospitals in the United Kingdom reported VR diabetes training led to clinicians having increased level of confidence in managing diabetes emergencies.^22^ After using VR, confidence increased in diabetes emergency management.^21^ Diabetes AR has also shown benefit for clinicians and patients, being portable and useable on a wide variety of smart devices, AR diabetes tools can provide useful information for decision-making support related to real-life scenarios.^25^

For VR patient education of diabetes, VR simulators provide the immersive platform on which to deliver real-world scenarios and information for training patients in self-management behaviors which can be experienced anywhere, anytime in a safe training environment without risk to the patient.^25^ It is important than when diabetes VR scenarios are being developed for patients such as training, the scenarios should be as close as possible to the real-life individual’s lived experience with diabetes.^25^

Training children with diabetes has been achieved with VR and AR,^27^ especially by integrating medical information and advice into games^29-31^ to enhance learning on an enjoyable immersive platform. The VR training for children shows promise to boost understanding of the disease and self-management skills.

For treating and managing diabetes, VR applications include assisting with carb counting, advice and logging of insulin, encouraging exercise, coaching in lifestyle improvements, and VR telehealth consultations. Although VR has shown advantages as a new opportunity, through automation and immersion, with many opportunities for VR and AR in diabetes management, using these technologies for diabetes is still in infancy.^25^

Rule-based simulator models are commonly used to mathematically model insulin responses, closed loop and clinical impact of diabetes conditions.^45^ A leading example is the U.S. Food and Drug Administration (FDA)-accepted UVA/Padova metabolic simulator,^46^ in use for over a decade. This UVA/Padova simulator model has also been useful to evaluate new designs of artificial pancreas algorithm, aiming to improve exercise safety in T1D.^47^ These diabetes simulator models are another area which can in future be more closely linked to VR. This could increase the accuracy of diabetes simulators within VR. These diabetes metabolism simulators are outside the main scope of this article, but a separate review of recent advances is warranted to identify their potential for VR integration.

Precursors to VR diabetes include online web-app learning modules with video content for diabetes topics.^48^ Evaluations have shown high satisfaction with such online web apps, platforms, and integrated diabetes video learning modules. Combining this multimedia diabetes content^48^ with NHS supported online training modules for adults with diabetes,^16^ and cine-VR 360 video,^17^ demonstrates potential for innovative VR modules to integrate diabetes learning.^49^

When future VR systems are being developed for diabetes, they should include a wide range of stakeholder inputs, including from people living with diabetes, pharmacists, practitioners, and diabetes educators. To gather such wide input, digital platforms could add value such as VR diabetes common accounts or addresses such as Facebook groups, Slack accounts or Github accounts for all those interested in VR diabetes to participate. As the possibilities of using VR in diabetes are so widespread and promising, this will provide direction and foundation for future VR tools in diabetes, which are expected to promise enhanced training of diabetologists and improvement in management and treatment for adults and children with diabetes.
